# Extract of a Letter from Mr. Blount to Mr. Summers, of Bond Street

**Published:** 1801-08

**Authors:** J. Blount

**Affiliations:** Birmingham


### *Extract of a Letter from Mr. Blount to Mr. Summers, of Bond Street.*

I HAVE enclofed you the copy of my letter to Dr. Jenner, refpecting the children inoculated at the Afylum; this you may ufe as you think proper.

Our Inoculation at the Difpenfary goes on very flowly, but with unvaried fuccefs. We are particularly attentive to ufe tranfparent fluid only. We alfo have found two, if not three cafes, to whom we have not been able to communicate either Small or Cow-pox. My private inoculations do not exceed thirty; and my five brethren may have done as much. Our numbers at the Difpenfary are a few fhort of 300; of thefe we have heard nothing unpleafant. I have examined three reports of Small-pox fucceeding the Cow-pox inoculation; the refult of thefe is highly in its favour, not one had the leaft foundation.

DEAR SIR,

WITH the Cow-pox matter you was fo obliging to fend me by my friend Mr. Samuel Rudder, I inoculated, April 17th, four children, viz.

John Lloyd, aged 2 years.

Maria Joyce, 2 years.

Twins { John Foxall, 2½ years.

Thomas Foxall, 2½ years old.

Of thefe, the inoculation of John Foxall only fucceeded. I fhall not trouble you with the daily progrefs of the inoculated part; only fhall obferve, that in this cafe, as well as the five fucceeding ones, the appearance in the arms was exactly what you have defcribed in your firft publication upon this interefting fubject.

1779, April 24. Thomas Foxall and Maria Joyce were again inoculated with tranfparent fluid, taken from the arm of John Foxall, both of which took effect.

May 2d. John Lloyd was inoculated with tranfparent fluid from the arm of Maria Joyce; this fucceeded.

May 10th. * * * * * was inoculated under the fame circumftance from John Lloyd.

May 18th. * * * * was inoculated from the arm of * * *.

Not one of thefe children had the leaft apparent conftitutional indifpofition. I could not difcover any fymptom of fever, nor had any one of them any eruption whatever. Their arms got well as foon as ufual under the Small-pox Inoculation. The only difference I could obferve was, that in every one of of them, the inflammation ſeemed to be more phlegmonous, if I may ſo expreſs myſelf. Theſe children make part of a family of 300. The houſe in which they live is about a mile diſtant from Birmingham. Every attention is paid to cleanlineſs and ventilation; ſo that I think the ſame effects may be expected as you uſually find in the country.

From *** I took ſome matter upon thread, with which I inoculated three children, all at the breaſt, about ſix months old, paupers in the crowded poor-houſe in a cloſe part of Birmingham; two did not take effect, and the third was taken to a diſtance by the mother the day after inoculation, ſo that I never ſaw the child after; but I have ſince heard, that the mother returned again in a few days with the child, who had about fifty eruptions, of what ſhe thought was the *Small-pox*. The diſeaſe appearing ſo ſlight, ſhe did not think it neceſſary to trouble me with it; thus I loſt the only opportunity of ſeeing the diſeaſe in this form, and of making any remark on the peculiarity of its appearance.

Auguſt 1.—I inoculated theſe ſix children with freſh crude variolous matter, but without effect.

25th.—I again repeated the variolous inoculation with the like reſult; and I intend to repeat it again as ſoon as I can procure ſome proper matter for the purpoſe.

When I have an opportunity of repeating the vaccine inoculation, which I hope to have ſoon, I will take the liberty of applying to you for ſome matter.

I am, &c.

*Birmingham.* J. BLOUNT.

June 14, 1801. Since the above was ſent to Dr. Jenner, theſe ſix children were put to ſleep with a child who had the Small-pox in the natural way, and not one took the diſeaſe.

